# D,L‐3‐hydroxybutyrate in the treatment of glucose transporter 1 deficiency syndrome (Glut1DS)

**DOI:** 10.1002/jmd2.12461

**Published:** 2025-01-16

**Authors:** Aya Amer, Kathryn Murrell, Liza Edmonds, Isaac Bernhardt, Rhonda Akroyd, Bryony Ryder, Callum Wilson, Emma Glamuzina

**Affiliations:** ^1^ Dunedin Hospital Te Whatu Ora Health New Zealand, Southern Dunedin New Zealand; ^2^ Adult and Paediatric National Metabolic Service Starship Children's Hospital, Te Toka Tumai, Te Whatu Ora Health New Zealand Tāmaki Makaurau Auckland New Zealand; ^3^ Te Tātai Hauora o Hine Victoria University Wellington New Zealand

**Keywords:** cognitive impairment, D,L‐3‐HB, D,L‐3‐hydroxybutyrate, Glut1, glut1ds, ketogenic, ketones

## Abstract

**Background:**

Deficiency of the Glut1 transporter due to mono‐allelic variants in *SLC2A1* causes hypoglycorrhachia, resulting in a neurological spectrum from neonatal epilepsy to adult‐onset paroxysmal movement disorders (PMD). The brain utilises ketone bodies as an alternative energy source to glucose. Thus, early initiation of the ketogenic diet (KD) is standard care for Glut1 deficiency syndrome (Glut1DS). Commencement and adherence in older Glut1DS patients is difficult to achieve, leaving few treatment options. Oral D,L‐3‐hydroxybutyrate (D,L‐3‐HB) crosses the blood–brain barrier, making it a potential treatment for Glut1DS.

**Methods:**

A retrospective case review of patients with Glut1DS under the Adult and Paediatric National Metabolic Service (APNMS) of New Zealand, treated with D,L‐3‐HB between 2012 and 2023 was performed. Clinical notes, standardised, neuropsychological assessments and subjective data on and off D,L‐3‐HB were obtained. The best on and off D,L‐3‐HB measures of working memory (WMI) and processing speed (PSI) were compared to assess the efficacy.

**Results:**

D,L‐3‐HB was offered to 12 patients with Glut1DS (age 10–52 years). Compliance‐dependent improvements in subjective, cognitive and adaptive function were reported by those who were reassessed on‐treatment (9/12). Four reported improved PMD. Objective improvements were found in WM (9/9) and PS (6/9). Subjective improvements were reported in patients' health, wellbeing and independence.

**Conclusions:**

KD remains standard of care for Glut1DS, but effective alternatives are lacking for those who do not tolerate this. D,L‐3‐HB was associated with improved WM, PS and perceived life quality in this small group of patients with Glut1DS, thus providing a potential treatment for this distinct group.

Synopsis

Exogenous ketones (EK) may improve working memory (WM) and processing speed (PS) in older Glut1 deficiency syndrome (Glut1DS) patients who cannot tolerate or establish a ketogenic diet (KD).

## 1 INTRODUCTION

Glucose transporter 1 deficiency syndrome (Glut1DS) is a partially treatable, neuro‐metabolic disorder, resulting from mono‐allelic variants in *SLC2A1* (MIM*138140).^1^ It has an estimated prevalence of 1 in 90 000. The Glut1 transporter transfers glucose across the blood–brain barrier and red cell membranes. Glut1 transporter deficiency leading to human disease, was described by De Vivo et al. in 1991. Two neonates presented with epilepsy, hypoglycorrhachia, low cerebrospinal fluid (CSF) lactate, developmental delay and decreased density of binding sites for the Glut1 transporter. Seizures resolved on ketogenic diet (KD).^2^ Over time, a broader phenotypic spectrum emerged, ranging from infantile‐onset ataxia, static motor delay, paroxysmal movement disorders (PMD) to asymptomatic. Intellectual disability (ID) in adult cohorts is common, but exercise‐induced dyskinesia or dysarthria may be the only symptom.^1^

Monocarboxylates including lactate and ketone bodies enter the central nervous system via the MCT1 transporter, providing an alternative energy source.^3^ KD improves seizures, PMDs and cognition, and remains the standard of care in Glut1DS.^4^, ^5^ Implementation and maintenance of the KD in adolescents and adults is challenging, and frequently infeasible.^6^, ^7^ Alternative evidence‐based, disease‐specific treatments for Glut1DS are limited.^4^ A high‐carbohydrate, low glycaemic index diet with regular intake of uncooked corn‐starch (UCCS) is frequently utilised, however this lacks objective evidence of benefit.^8^ Acetazolamide has been utilised to treat PMD in a small case series.^9^, ^10^, ^11^ Triheptanoin, an odd‐chain triglyceride with anaplerotic potential,^12^ showed promise in case series,^13^, ^14^ however, a subsequent randomised double‐blinded trial failed to demonstrate improved seizure frequency.^15^ Sodium lactate infusion was utilised to treat seizures in two patients, providing proof of principle that lactate may have positive therapeutic effects in Glut1DS.^16^

Interest in exogenous ketones (EK) is growing over multiple fields of research. A racemic mixture of D,L‐3‐hydroxybutyrate (D,L‐3‐HB) was first used to treat leukoencephalopathy and cardiomyopathy in multiple acyl‐CoA dehydrogenase deficiency (MADD) in 2003.^17^ D,L‐3‐HB is now utilised in ketone‐deficient metabolic disorders as standard of care. Studies in healthy individuals showed that D,L‐3‐HB generated sustained elevation of total β‐hydroxybutyrate in blood, suggesting EK could be a viable alternative to KD.^18^ However, key differences between nutritional ketosis and EK exist, with better tolerance, more rapid ketosis, but lower levels of metabolic adaptation in the latter.^19^

We present a retrospective review of a cohort of genetically confirmed Glut1DS patients, managed by the New Zealand Adult and Paediatric National Metabolic Service (APNMS), and commenced on oral D,L‐3‐HB in an attempt to improve cognition and PMD.

## 2 METHODS

Glut1DS patients cared for by APNMS were identified in the clinical service database. Twelve patients between 2012 and 2023 were identified as unable to adhere to or tolerate KD, and were offered oral D,L‐3‐HB, at 500 mg/kg/day divided four hourly during waking hours as part of clinical care. Clinical records including email correspondence and comprehensive neuropsychology reports, prescribing data, patient‐reported side effects, any reports at clinic of PMD or seizures, patient and family subjective reports were obtained and reviewed.

**FIGURE 1:**
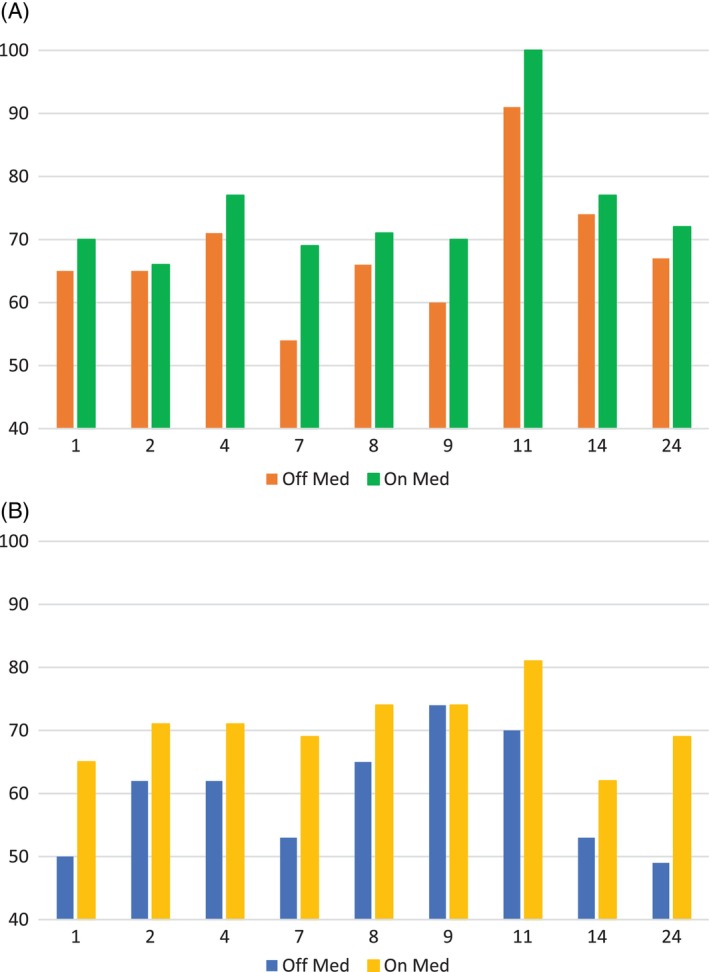
Graphs of Working Memory Index (A) and Processing Speed Index (B) per patient On and Off D,L 3‐hydroxybutyrate (normal being 100 with standard deviation ± 15).

Ethics approval for this retrospective review, and for the D,L‐3‐HB treatment was not sought. This was not a clinical trial and was considered a safe treatment used for other inherited metabolic disorders (IMD). Patients and caregivers verbally consented to D,L‐3‐HB.

Funding for D,L‐3‐HB was obtained through PHARMAC, a New Zealand government agency responsible for the funding of medicines, using the Named Patient Pharmaceutical Application. This is a formal pathway to obtain treatment access for patients with exceptional clinical circumstances. Objective evidence of improvement in Cognitive Proficiency (CP) measures was required for funding re‐approval. Accordingly, assessments were performed by the APNMS registered clinical neuropsychologist using the CP measures available in the standardised age‐appropriate neuropsychological assessments (WISC‐IV, WAIS‐IV).

To determine mean and statistical significance of change, standardised scores of CP, specifically working memory (WMI) and processing speed (PSI), ‘best on’ versus ‘best off’ performance, were analysed based on within‐subject comparison using Microsoft Excel's (2018) one tailed, paired t‐test.

## 3 RESULTS

### 3.1 Glut1DS cohort

Twenty‐four patients were identified in the APNMS clinical patient database to have a biochemical and molecular diagnosis of Glut1DS. At review, patient ages ranged from 2 to 59 years, with a median age of 14 years. Age at diagnosis ranged from 10 weeks to 52 years. Clinical presentation reflected the full phenotypic spectrum; one patient was diagnosed after a classical neonatal presentation, while 16/24 presented with absence seizures in the first 10 years. Ten (10/24) patients had PMD, including six with exercise‐induced dyskinesia and one with alternating hemiplegia of childhood. Five of the cohort (5/24) did not have a diagnosis of ID.

### 3.2 D,L‐3‐HB treatment cohort

Twelve patients were identified by the APNMS as needing treatment, but unable to tolerate or commence KD. All had neuropsychometric assessments at baseline as part of routine care. Eleven (11/12) had intellectual disability with mean full‐scale IQ 68 (range 40–88). On commencement of D,L‐3‐HB ages ranged from 10 to 50 years. Nine (9/12) had infrequent (less than one per year) seizures (absence, generalised tonic–clonic), 8/12 had PMD and 7/12 had mental health or behaviour issues (Table 1). Nine (11/12) were not on any Glut1DS‐specific treatment at the time of starting. Two patients were on regular UCCS prior to and after commencing D,L‐3‐HB. One (Patient 14) had been on KD for 15 years, but experienced multiple complications including weight loss and low bone mineral density. He found the diet limiting and was not achieving ketosis.

**TABLE 1:** Clinical data of patient cohort with Glut1DS who trialled D,L 3‐hydroxybutyrate.

Patient ID	Gender	Diagnosis (*SLC2A1* Genotype)	Seizures	PMD	Mental health or behaviour issues	Intellectual disability (IQ < 70)	Adherence	Subjective reports of improvement	Excluded
1	Female	c.1234 T > C (P.Trp412Arg)^a^	Yes (weekly absence)	yes	Yes	Yes	4 hourly in the day, very regular scripts	Yes, more alert, improved focus	
2	Male	c.277C > T (p.Arg93Trp) (3‐O‐Methyl‐D‐Glucose 53% controls 2009)	No	Yes	No	Yes	4 hourly in the day, very regular scripts	Yes, more alert, improved focus, less PMD. Extinguishes PMD if taken	
3	Female	c.464C > T (p.Ala155Val)	Yes (weekly absence)	Yes	Yes	Yes	No	Did not feel it helped	Yes
4	Male	c.476 T > G p.(Leu159Arg))	Yes	No	Yes	Yes	Limited use	More alert, improved focus, forgets to take it	
7	Male	c.464C > T (p.Ala155Val)	No	Yes	Yes	Yes	Intermittent use, irregular prescribing	Yes, more alert, improved focus	
8	Female	c.464C > T (p.Ala155Val)	Yes (stable on lamotrigine)	No	Yes	Yes	No, declined treatment	Could not tolerate taste	
9	Male	c.464C > T (p.Ala155Val)	Yes (<1/ year)	No	Yes	Yes	Limited use, irregular prescribing	More focussed, improved speech, did not like taste	
11	Male	c.1075G > T (p.Glu359^a^)	No	Yes	No	No	Intermittent use. Uses when working and exercising	More focussed, improved speech	
13	Male	1p34.2 microdeletion including *SLC2A1*	Yes	Yes	Yes	Yes	Intermittent use, irregular prescribing	School and patient report improved focus and speech	Yes
14	Male	c.381‐382delTC^a^	Yes	Yes	No	Yes	4 hourly in the day, very regular scripts	Greatly improved quality of life compared to KD. Improved focus.	
17	Female	c.115‐2A > T	Yes	Yes	No	Yes	No		Yes
24	Female	c.3541G > A (p.Gly181Ser)	Yes	No	Yes	Yes	4 hourly in the day, regular scripts	Improved behaviour, more alert, ongoing occasional seizures	
Total			9	8	7	11	4 fully compliant		3

Abbreviations: IQ, intelligence quotient; PMD, paroxysmal movement disorder.

^a^ Research lab.

### 3.3 Monitoring, neuropsychological assessment and analysis

#### 3.3.1 Objective measures

Nine of 12 patients completed both on and off D,L‐3‐HB assessments. Three patients were unable to complete on‐medication psychometric testing and were excluded. Statistically significant differences were found between pre‐ and post‐treatment scores for WMI and PSI. Eight (8/9) patients had improved PSI score on treatment. All nine improved their WMI score, though Patient 2 improved by only one point. Three patient's best on and off scores spanned the WISC‐IV and WAIS‐IV (Table 2, Figure 1).

**TABLE 2:** Psychometric data per patient on and off treatment with D,L 3‐hydroxybutyrate.

Patient ID	Age off (years)	Age on (years)	Off test	On test	WMI OFF	WMI ON	PSI OFF	PSI ON
1	13.16	14.51	WISC IV	WISC IV	65	70	50	65
2	12.83	18.71	WISC IV	WAIS IV	65	66	62	71
4	17.28	17.58	WAIS IV	WAIS IV	71	77	62	71
7	16.53	21.90	WISC IV	WAIS IV	54	69	53	69
8	18.77	24.02	WAIS IV	WAIS IV	66	71	65	74
9	50.78	51.63	WAIS IV	WAIS IV	60	70	74	74
11	15.59	20.70	WISC IV	WAIS IV	91	100	70	81
14	17.87	18.90	WAIS IV	WAIS IV	74	77	53	62
24	10.89	13.49	WISC IV	WISC IV	67	72	49	69
				**Average**	68.11	74.67	59.78	70.67
				***P*(*T*≤*t*) one‐tail**		0.00177		0.00022

Abbreviations: PSI, processing speed index; WMI, working memory.

#### 3.3.2 Subjective reports

Improvements in daily function on treatment were reported by patients, parents and carers at clinic appointments or via email. Patients were noted to be more oriented to their environment and clearer in their thinking, describing a feeling of ‘pieces of the jigsaw puzzle coming together’. Carers observed improved independence, motivation and focus, describing patients on treatment as ‘being able to stay on task despite distractions’, being able to cook, help out with household chores and do better at school and work. There were also reported improvements in patients' mental health. They were described as more able to engage with therapy and cope better with stress. They de‐escalated quicker, were less moody and less fatigued. The clinical team could observe improved speech and speed of answering questions after taking D,L‐3‐HB in the clinic room.

#### 3.3.3 Seizures and PMD

Pre ‘on treatment’ diaries were not kept. There was no reported change in seizure frequency, though the nine patients assessed on treatment had no or infrequent seizures. All five patients who had PMD reported fewer ‘wobbly events’ at clinic visits. One patient reported that when the events occurred, they were terminated through administration of D,L‐3‐HB.

#### 3.3.4 Adherence

Adherence record was based on carer and patient report, as well as prescription data. Four patients took the D,L‐3‐HB as prescribed, two had limited use but found it beneficial and three used it when needed, particularly if they were working under stress, or needed to improve task performance. Despite significant ID, the four patients who took it every day knew exactly when it was due and when they needed it. Despite worldwide shortages, D,L‐3‐HB has been consistently available for these patients. Over the course of treatment from 2012 to 2023, the price has increased substantially. We are not privy to what this price is.

#### 3.3.5 Side effects

There were no reported D,L‐3‐HB related adverse events. Sodium, renal function and blood pressure monitoring occurred ad hoc but when measured were in normal range.

## 4 DISCUSSION

Glut1DS caused by mono‐allelic variants in *SLC2A1* is a brain energy disorder that results in a wide spectrum of neurological manifestations. We present the data on nine patients ranging between 10 and 50 years, treated with D,L‐3‐HB as part of clinical care. To our knowledge, this is the first report of positive objective and subjective neuropsychological outcomes, in particular improved CPI measures, daily performance and interaction. This is a retrospective case review of the real‐world experience of caring for such patients, and as a result has many limitations.

These positive findings are different to previously reported experience. An earlier, smaller case series, examining the use of D,L‐3‐HB in Glut1DS as a partial or total progressive substitution to KD in three patients aged 11 to 22, noted a clinical deterioration with increased frequency of seizures and myoclonus.^20^ This suggests that the efficacy of D,L‐3‐HB may be inferior to KD. In contrast, albeit in rats, a subsequent laboratory study published in 2020 investigated the effect of EK on motor performance and blood ketone levels (in the form of R‐beta‐hydroxybutyrate) in rodent models with and without Glut1DS. The use of ketone esters resulted in long‐term improvement in motor function, while simultaneously increasing the degree of blood ketosis.^21^, ^22^ There are no publications looking at the use of D,L‐3‐HB in older patients with Glut1DS, and the 2020 consensus guideline concludes there is no evidence for the use of ketone bodies in Glut1DS.^4^

Deciding on the best end‐point score to use when assessing patients for ongoing PHARMAC funding of D,L‐3‐HB was based on normal clinical practice, with some consideration of the sensitivity of measures that were available. A measure of this in neurocognitive assessment is the CPI, which is a combination of WMI and PSI in the 2003 WISC‐IV and WAIS‐IV. CPI scores serve as an important marker of neurocognitive status.^23^ We found statistically significant improvement in performance on these CPI measures in these patients. The impact of D,L‐3‐HB treatment was also noted in the subjective reports. Patients initially reported feeling awake and ‘sharper’, like a ‘fog had lifted’, suggesting improved ability to focus and attend to tasks.

The choice of D,L‐3‐HB and the dose was based on that used to treat MADD^17^ and other IMDs. The ideal treatment regimen however is not known, particularly in Glut1DS. van Rijt et al^24^ looked at enantiomer‐specific pharmacokinetics of D,L‐3‐HB in rats and MADD patients. Both D‐3‐HB and L‐3‐HB enantiomers peaked at 30–60 min, and approached baseline after 3 h. They recommended a dosing schedule of six to eight times a day, tailoring to the individual patient ketosis. Accordingly, administering higher or more frequent doses of D,L‐3‐HB in our cohort could have resulted in a more sustained or greater objective and subjective improvement. In the ‘real world’, this would have been cost‐prohibitive and potentially associated with poorer adherence. Given the shortage of D,L‐3‐HB faced in some regions of the world, and the proliferation of ‘ketones’ available in the health food market at substantially lower cost, understanding the optimal ratio of the D‐3‐HB and L‐3‐HB enantiomers, which may differ depending on the target organ,^22^, ^24^ hepatic metabolism, transport into the CNS, and utilisation in brain is of interest. Measurement of β‐hydroxybutyrate following D,L‐3‐HB administration in blood and CSF may have been informative.

### 4.1 Limitations

We present our real‐world experience of the use of D,L‐3‐HB in patients with Glut1DS. This is not a clinical trial and was not ‘designed’. Patients were essentially started on treatment with consent in a clinical setting. The only boundary to treatment was to assess objective CPI measures to achieve ongoing government funding. Accordingly, most efficacy data available is subjective and non‐standardised. The subjective treatment outcomes, and measures of PMD frequency would have been better assessed with formalised questionnaires and symptom diaries.

A limitation of the objective measures of CPI was that assessments on‐treatment were not taken at a standard time post‐dose. In addition, the psychologist was not blinded to treatment conditions. This should not have biased post‐assessment scores in favour of the medication as the Wechsler Assessment tools are validated means of assessing cognitive abilities, performed by the participant. Further, due to the assessment age cut‐offs, three patients were assessed using WISC‐IV off, and WAIS‐IV on. This change has the potential to falsely elevate the impact of treatment as WAIS‐IV may score higher.^25^ This highlights the need for future research planning to ensure that end‐point measures are consistent over time.

Finally, there were no reported D,L‐3‐HB‐related adverse events in our treatment group; however, we could have had more data regarding the blood ketone levels, blood pressures and sodium status in this cohort.

As this is a retrospective descriptive review rather than a formal clinical trial, our results do not have the necessary power to change clinical practice. Nonetheless, due to the rarity of the condition, and this being a relatively large cohort, it can serve as a starting point for future work looking into whether D,L‐3‐HB can indeed be used as a treatment option for Glut1DS patients, with or without other less invasive interventions such as UCCS and/or a low GI diet. ‘Trials of therapy’ are very common practice in rare disorder medicine. The limitations acknowledged here exemplify the need to have a more ordered approach to monitoring patients who commence such treatments. Engaging a formal monitoring plan would allow for more accurate data review over time and improve sharing of information.

## 5 CONCLUSION

KD remains the standard of care for Glut1DS, but effective alternatives are lacking for those who do not tolerate this intensive diet. Use of D,L‐3‐HB in older Glut1DS patients was associated with improved WMI and PSI, as well as subjective measures of improved quality of life and independence. This is the first report suggesting benefit of EK to treat some of the non‐seizure related manifestations of Glut1DS. Future research, including formalised placebo‐controlled trials and more robust end‐point analysis, is required to further delineate the role of EK in the treatment of Glut1DS.

## AUTHOR CONTRIBUTIONS

Aya Amer: wrote the primary manuscript. Kathryn Murrell: performed the neurocognitive assessments, data analysis and manuscript writing. Liza Edmonds: Supervision of Aya Amer and guidance in writing. Isaac Bernhardt: Patient care and editing. Rhonda Akroyd: Dietetic management and advice, editing. Bryony Ryder: Patient care and editing. Callum Wilson: Initial inception of plan to use this therapy. Patient care and editing. Emma Glamuzina: Patient care, management, study design, writing and supervision of Aya Amer.

## FUNDING INFORMATION

Funding for the D,L‐3‐hydroxybutyrate and any travel to Auckland from around the country was obtained from NZ government via Te Pātaka Whaioranga PHARMAC and Te Whatu Ora Health New Zealand as part of routine care. The authors confirm independence from the sponsors and the content of the article has not been influenced by the sponsors.

## CONFLICT OF INTEREST STATEMENT

Aya Amer, Kathryn Murrell, Liza Edmonds, Isaac Bernhardt, Rhonda Akroyd, Bryony Ryder, Callum Wilson, and Emma Glamuzina have no conflict of interest to declare.

## ETHICS APPROVAL AND INFORMED CONSENT

No ethics approval was sought as this nutritional product was given as a part of routine medical care and considered low risk. As this had not been given previously for this indication, all patients and family verbally consented to this therapy. All procedures followed were in accordance with the Helsinki Declaration of 1975, as revised in 2000.

## ANIMAL RIGHTS

Not applicable—this article does not contain any studies with animal subjects performed by any of the authors.
